# The Binding of BF-227-Like Benzoxazoles to Human α-Synuclein and Amyloid β Peptide Fibrils

**DOI:** 10.1177/1536012118796297

**Published:** 2018-09-14

**Authors:** Lee Josephson, Nancy Stratman, YuTing Liu, Fang Qian, Steven H. Liang, Neil Vasdev, Shil Patel

**Affiliations:** 1 MedChem Imaging, LLC, Boston, MA, USA; 2 Division of Nuclear Medicine and Molecular Imaging, Department of Radiology, Massachusetts General Hospital, Harvard Medical School, Boston, MA, USA; 3 Biomarkers Preclinical Imaging and Pharmacology, Research and Early Development, Biogen, MA, USA; 4 Biologics Drug Discovery, Biogen, Cambridge, MA, USA; 5 Translational Imaging Engine, Eisai AiM Institute, MA, USA. Vasdev is now with Azrieli Centre for Neuro-Radiochemistry, Centre for Addiction and Mental Health and Department of Psychiatry, University of Toronto, Toronto, Ontario, Canada

**Keywords:** α-synuclein, amyloid β peptide, binding affinity, fibrils, BF-227, PET

## Abstract

Development of an α-synuclein (α-Syn) positron emission tomography agent for the diagnosis and evaluation of Parkinson disease therapy is a key goal of neurodegenerative disease research. BF-227 has been described as an α-Syn binder and hence was employed as a lead to generate a library of α-Syn-binding compounds. [^3^H]BF-227 bound to α-Syn and amyloid β peptide (Aβ) fibrils with affinities (K_D_) of 46.0 nM and 15.7 nM, respectively. Affinities of BF-227-like compounds (expressed as K_i_) for α-Syn and Aβ fibrils were determined, along with 5 reference compounds (flutafuranol, flutemetamol, florbetapir, BF-227, and PiB). Selectivity for α-Syn binding, defined as the K_i_(Aβ)/K_i_(α-Syn) ratio, was 0.23 for BF-227. A similar or lower ratio was measured for analogues decorated with alkyl or oxyethylene chains attached to the oxygen at the 6 position of BF-227, suggesting a lack of involvement of the side chain in fibril binding. BF-227-like iodobenzoxazoles had lower affinities and poor α-Syn selectivity. However, BF-227-like fluorobenzoxazoles had improved α-Syn selectively having K_i_(Aβ)/K_i_(α-Syn) ranging from 2.2 to 5.1 with appreciable fibril affinity, although not sufficient to warrant further investigation. Compounds based on fluorobenzoxazoles might offer an approach to obtaining an α-Syn imaging agent with an appropriate affinity and selectivity.

## Introduction

Development of an α-synuclein (α-Syn) selective, positron emission tomography (PET) imaging agent for early diagnosis and evaluation of therapies in Parkinson disease is one of the most sought after goals in neurodegenerative disease research.^1-3^ Fluorine-18-labeled BF-227 (2-(2-[2-dimethylaminothiazol-5-yl]ethenyl)-6-(2-[fluoro]ethoxy)benzoxazole) was first used as a PET tracer for amyloid β peptide (Aβ) plaques, with a reported K_D_ for synthetic Aβ fibrils of 4.1 nM.^4^ Later, it was determined that BF-227 was not selective and bound to both synthetic Aβ fibrils (K_D_ = 1.31 nM) and synthetic α-Syn fibrils (K_D_ = 9.3 nM).^5^ Histochemical analysis with a high concentration (100 μM) of BF-227 led to Lewy body fluorescence.^5^ However, subsequent studies indicated [^18^F]BF-227 failed to bind to α-Syn-positive, Aβ-negative dementia with Lewy body human brain homgenates and lacked a sufficient affinity or selectivity to serve as an α-Syn imaging agent.^3^ Since BF-227 has been described as having a high affinity for pathological forms of α-Syn, we were interested in using its scaffold as a lead molecule to create a focused library of BF-227-like compounds with the following objectives: (1) to further characterize the interaction of BF-227 with α-Syn and Aβ fibrils and (2) to identify compounds with improved binding affinity and selectivity for α-Syn fibrils. The affinities of the BF-227-like compounds and reference compounds were determined for reconstituted α-Syn and Aβ fibril preparations. Reference compounds were 5 compounds used clinically to image Aβ (flutafuranol, flutemetamol, florbetapir, BF-227, and PiB), Thioflavin S, benzotriazole-1 (BTA), 1,4-bis(paminostyryl)-2-methoxy benzene (BMB)-1, Clorglyine, and RO-16-6491. The rationale for reference compound selection is given subsequently.^3^

## Materials and Methods

### Compound Sources

**Figure 1.**
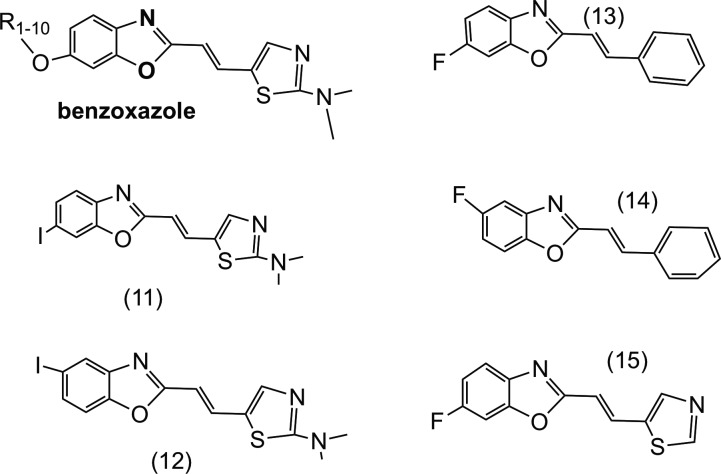
Structure of BF-227-like compounds. Structures 1-10 are benzoxazole derivatives with R, a fluoroethyl group, with varying length hydrocarbon chains or oxyethylene groups appended (see Table 1 for individual compound designations). The structure of compound (1) BF-227 is shown in Figure 2.

**Figure 2.**
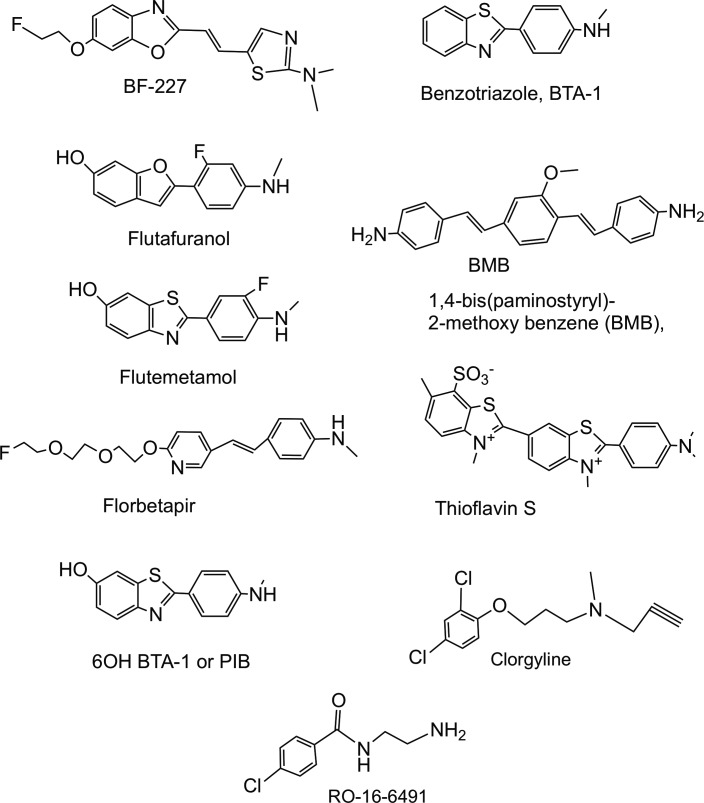
Structures of reference compounds.

BF-227-like compounds (Figure 1) were provided by MedChem Imaging LLC (Boston, Massachusetts). Reference compounds (Figure 2) obtained from Sigma-Aldrich (St. Louis, Missouri) were BTA-1, BMB, clorgyline, and Thioflavin S; from WuXi Pharma Tech (Shanghai, China) were BF-227, florbetapir (AV-45), and flutafuranol (NAV 4694); from ABX GmbH (Radeberg, Germany) 6-OH-BTA1 (PiB); from Santa Cruz Biotechnology (Dallas, Texas) RO-16-6491; from GE Healthcare (Oslo, Norway) flutemetamol. Radiolabeled [^3^H]BF-227 (66 Ci/mmole; 1 mCi/mL) was from Vitrax Radiochemicals (Placenta, California). Compound structures are provided (Figures 1 and 2) with molecular properties (MW, tPSA, LogP, CLogP) determined using Chemdraw 16.01.04 (Tables 1 and 2).

**Table 1.** BF-227-Like Compound Affinity (Ki, nM) for α-Synuclein and Amyloid β Fibrils.

#	R (Side Chain)	MW	n	α-Syn Ki	AβK_i_	K_i_(Aβ)/K_i_(α-Syn)	Log *P*	tPSA	CLogP
1	FCH_2_ (CH_2_)O-	333.38	6	53 (46; 60)	12 (11; 13)	0.23	2.94	46.42	2.59
2	FCH_2_ (CH_2_)_2_O-	347.41	3	157 (126; 188)	9.01 (3; 14)	0.06	3.05	46.42	2.82
3	FCH_2_ (CH_2_)_3_O-	361.44	3	378 (306; 451)	31 (25; 36)	0.08	3.50	46.62	3.20
4	FCH_2_ (CH_2_)_4_O-	375.46	3	297 (222; 371)	28 (24; 31)	0.09	3.92	46.42	3.73
5	FCH_2_ (CH_2_)_5_O-	389.49	3	462 (439; 486)	23 (20; 27)	0.05	4.34	46.42	3.79
6	FCH_2_ (CH_2_)_6_O-	403.52	3	212 (163; 260)	27 (25; 29)	0.13	4.75	46.42	4.79
7	FCH_2_[O-CH_2_-CH_2_]O-	377.43	3	83 (79; 88)	11 (10; 12)	0.13	2.79	55.65	2.34
8	FCH_2_[O-CH_2_-CH_2_]_2_O	421.49	3	52 (33; 73)	13 (12; 15)	0.25	2.63	64.88	2.20
9	FCH_2_[O-CH_2_-CH_2_]_3_O-	465.54	3	100 (84; 117)	26 (20; 31)	0.26	2.47	74.11	2.07
10	FCH_2_[O-CH_2_-CH_2_]_4_O-	509.59	3	75 (59; 92)	18 (16; 20)	0.24	2.32	83.34	1.94
Iodo benzoxazoles									
11	Not applicable	397.23	3	74 (382; 1110)	223 (188; 259)	0.30	4.23	37.19	3.54
12	Not applicable	397.23	3	276 (127; 426)	96 (94; 97)	0.35	4.23	37.19	3.54
Fluoro benzoxazoles									
13	Not applicable	239.25	3	328 (251; 405)	1247 (1079; 1414)	3.8	4.10	21.59	4.25
14	Not applicable	239.25	3	238 (178; 298)	528 (486; 570)	2.2	4.10	21.59	4.25
15	Not applicable	246.26	3	286 (255; 316)	1446 (1065; 1826)	5.1	2.34	33.95	2.40

**Table 2.** Reference Compound Affinity (K_i_ in nM) for α-Synuclein and Amyloid β Fibrils.

Compound	MW	n	α-SynK_i_	Aβ K_i_	K_i_ (Aβ)/K_i_ (α-Syn)	Log *P*	tPSA	CLogP
BF-227	333.38	6	53 (46; 60)	12 (11; 13)	0.23	2.94	46.42	2.59
Flutafuranol (NAV 4694)	258.25	5	32 (27; 38)	42 (34; 50)	1.34	2.82	53.85	2.28
Flutemetamol	274.31	3	48 (44; 53)	65 (60; 70)	1.35	3.44	44.62	2.96
Florbetapir (AV-45)	360.43	5	24 (19; 29)	12 (9; 15)	0.46	2.85	52.1	3.02
PiB	256.32	6	55 (51; 59)	77 (68; 87)	1.4	3.28	44.65	2.82
BTA-1	240.32	8	64 (61; 67)	146 (129; 163)	2.3	3.67	24.39	3.48
BMB	342.44	6	184 (109; 258)	76 (62; 91)	0.35	4.68	61.27	4.99
Thioflavin S	510.66	3	>9000	2150 (1865; 2435)	≤0.2	N.O.	N.O.	N.O.
Clorgyline	272.17	3	>9000	>5000	NO	3.35	12.7	4.31
RO-16-6491	198.65	3	>9000	>7000	NO	0.85	55.12	1.09
PET agent training set, mean (SD)		5				3.03 (0.28)	53.1 (11.2)	2.77 (0.26)

Abbreviations: BMB, 1,4-bis(paminostyryl)-2-methoxy benzene; BTA, benzotriazole; PET, positron emission tomography; SD, standard deviation.

### Preparation of Aβ Fibrils

Aβ_1-42_ peptide 5 mg, Cat #20276-5 (Anaspec, Fremont, California) was dissolved in 250 µL dimethyl sulfoxide (DMSO) for 2 hours with occasional swirling, followed by water bath sonication, (B-200 Branson [Danbury, Connecticut]) for 5 minutes. The clear peptide solution was transferred to a 15 mL conical tube. (Cat #430052, Corning [from Sigma-Aldrich]), and the original vial was washed with 625 µL of double deionized Milli-Q-H_2_O, which was then combined with 250 µL peptide/DMSO solution in the conical tube. Subsequently, 4 mL of Milli-Q-H_2_O was added to the conical tube followed by the addition of 125 µL of 1 M Tris–HCl, pH 7.5 with gentle mixing of the solution. The final volume was 5 mL, with the starting peptide concentration at 1 mg/mL. The peptide solution was divided into 5 × 1 mL aliquots with 1.5 mL Eppendorf tubes and incubated for 72 hours at 37°C with shaking at 1000 rpm in an Eppendorf Thermomixer. Fibril formation was confirmed by visual inspection for turbidity of the solution and further confirmation with Thioflavin T fluorescence spectroscopy (Sigma-Aldrich, Cat#T3516). Centrifugation at 15 000*g* for 15 minutes was performed to pellet fibrils, and the supernatant was assessed for protein, which was minimal by A280 or the BCA protein assay. The supernatant was discarded, and the pelleted fibrils were resuspended (1 mg/pellet) with phosphate-buffered saline (PBS; pH7.4) to obtain a stock concentration of 444 µM (expressed as a monomer equivalent). Fibril stock solutions were stored at −80°C.

### Preparation of α-Syn Fibrils

Human full-length α-Syn (NM_000345) was cloned into the ampicillin-resistant *Escherichia coli* expression vector PET7-7 and transformed into BL21 (D3) strains for expression. The recombinant α-Syn was expressed and purified to ≥95% purity as described previously.^6^ The monomeric α-Syn was formulated in 10 mM Tris–HCl, pH 7.6, 50 mM NaCl at 5 mg/mL. For fibril formation, 500 µL of purified α-Syn monomer was subjected to continuous shaking at 1000 rpm in an Eppendorf Thermomixer at 37°C for 7 days. The monomer was removed from fibrils by high-speed centrifugation with a Beckman-Coulter tabletop Optima MAX-XP (Beckman-Coulter, Indianapolis, Indiana) ultra-centrifuge at 600 000*g* for 40 minutes, followed by 3 PBS/centrifugation wash steps. After each centrifugation step, the supernatant was removed and the concentration of α-Syn in the supernatant was calculated from absorbance at 280 nm. The pellet was resuspended in PBS, and the concentration of fibrils was estimated by subtracting the total amount of α-Syn in the supernatant from all the wash steps and confirmed by BCA assay before use.

### Binding Studies With [^3^H]BF-227

Saturation binding of [^3^H]BF-227 was determined with increasing concentrations (1-400 nM) of radioligand and nonspecific binding obtained with 10 µM BTA-1, a reference agent structurally similar to imaging agent PiB. Competition studies were carried out using 5 nM [^3^H]BF-227. Experiments were performed in 50 mM Tris–HCl pH 7.5, 150 mM NaCl, and 0.1% bovine serum albumin in a reaction volume of 200 µL. Incubations were initiated with the addition of human α-Syn (0.5 mM/well) or Aβ_1-42_ (0.1 μM/well) fibrils at room temperature and terminated 2 hours later by rapid vacuum filtration over Whatman GF/C 96-well Unifilters (Brandel [Gaithersburg, Maryland]) presoaked in cold wash buffer (50 mM Tris–HCl, pH7.4), followed by four 200 µL washes with cold wash buffer. Filters containing bound ligand were mixed with 50 µL Microscint-PS (Perkin-Elmer [Waltham, Massachusetts]) and counted with a MicroBeta2 Scintillation Counter (Perkin Elmer). All data points were performed in triplicate. Values for the saturation binding dissociation constant (K_D_) and the maximal number of binding sites (B_max_) were determined by fitting the data to the equation Y = B_max_ × X/(X + K_D_) using nonlinear regression analysis. IC_50_ values were generated from the concentration–response curves using nonlinear regression analysis and converted to K_i_ values with the Cheng-Prusoff equation.^7^ Data analysis was performed with GraphPad Prism version 7.02.

## Results

Figure 1 and Table 1 show the structures of BF-227 and the BF-227-like compounds that were synthesized. For the BF-227 scaffold, the R group attached to the oxygen at the 6 position of the benzoxazole group was varied by increasing hydrocarbon chain length (#2 through #6, see Table 1) and by increasing the number of oxyethylene groups (#7 through #10, see Table 1). Two iodobenzoxazoles (#11, #12) and 3 fluorobenzoxazoles (#13, #14, #15), all lacking side chains, were also synthesized (Figure 1, Table 1). The benzoxazole ring of BF-227 and BF-227-like compounds is shown in bold.

The reference compounds used are shown in Figure 2. ^18^F or ^11^C isotopologues of 5 compounds (flutafuranol, flutemetamol, florbetapir, BF-227, and PiB) have been used to image Aβ by PET. While [^11^C]PiB and the Food and Drug Administration–approved [^18^F] versions of florbetapir, florbetaben, and flutemetamol have become important methods of analyzing neurodegenerative disease, these compounds have limitations because of a lack of correlation between amyloid deposition and disease stage and an inability to image nonfibrillar Aβ-plaques.^8^ Our reference thioflavin S is widely used as a histological stain for amyloid, fluorescing when bound to diverse types of fibrils including those of Aβ,^9^ α-Syn,^10^ and tau.^11^ Benzotriazole 1 is an uncharged compound structurally related to thioflavin used in the development of Pittsburgh compound B (PiB).^3^ A ^11^C isotopologue of BMB-1, a Congo red-like compound, has been used to image mylein basic protein.^12^ Clorglyine and RO-16-6491 are inhibitors of monamine oxidase A and monamine oxidase B, respectively, and were included because imaging agents designed to bind tau can bind to these targets.^13-15^

### Characterization of [^3^H]BF-227 Binding to α-Syn and Aβ Fibrils

**Figure 3.**
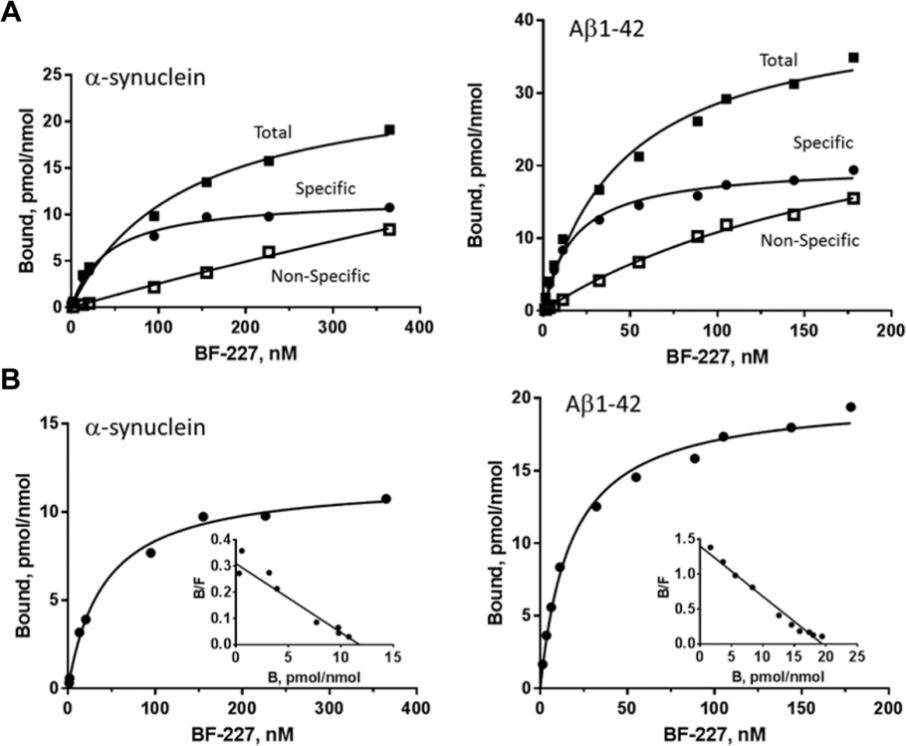
Binding of [^3^H]BF-227 to α-synuclein and amyloid β fibrils. A, The concentration dependence of [^3^H]BF-227 binding is shown, where specific = total − nonspecific binding. B, Binding isotherms and Scatchard plots from (A) are shown. Data were fit to a single-site binding model. Y-axis is pmoles tritiated tracer divided nmoles fibril (expressed as monomer).

The K_D_ values for the binding of [^3^H]BF-227 to α-Syn and Aβ fibrils were determined using filtration binding studies (Figure 3). The concentration dependence of specific and nonspecific binding to α-Syn and Aβ fibrils is shown in panel A. Nonspecific binding was defined by incubation with 10 μM BTA-1 and increased linearly with increasing [^3^H]BF-227 concentrations. Specific binding of [^3^H]BF-227 (specific = total − nonspecific) was fit to a single-site binding model using saturation and Scatchard plots (inset) to determine the binding affinity, K_D_ (Figure 3B). Individual K_D_ and B_max_ values are provided in Table S1 of the supplement. For α-Syn fibrils, a K_D_ of 46.0 (2.8) nM and a B_max_ of 12.7 (1.1) pmole/nmole was obtained and is expressed as the mean and standard deviation (within the parentheses), n=2. For Aβ fibrils, a K_D_ of 15.7 (3.1) nM and a B_max_ of 19.4 (2.7) pmol/nmol was obtained (mean [SD], n = 3) .

### Affinities of BF-227-Like Compounds for α-Syn and Aβ1-42 Fibrils

Table 1 shows binding affinties (K_i_ in nM) to α-Syn and Aβ fibrils for the 15 benzoxazole compounds shown in Figure 1. Results are expressed as inhibition constants (K_i_, nM) corrected for ligand occupancy.^7^ Each value is a geometric mean from the indicated numbers of experiments (n), with the numbers in parentheses indicating the low and high errors of the geometric mean. Also shown are the Log P, tPSA, and CLogP values for these compounds. These can be compared with means for the 5 Aβ imaging agents which can serve as an empirical training set to judge the likely in vivo behavior of new compounds.

### Affinities of Reference Compounds for α-Syn and Aβ Fibrils

Table 2 gives the affinties to α-Syn and Aβ fibrils for the reference compounds (Figure 2), along with their values of MW, logP, tPSA, and cLogP. Isotopologues of BF-227, flutafuranol, flumetamol, florbetapir, and PiB have been used to image Aβ by PET. All 5 bound both α-Syn and Aβ fibrils, with 2 (BF-227 and Florbetapir) exhibitng a preference for Aβ over α-Syn, with a values of 0.23 and 0.46 for the ratio of their K_i_’s (K_i_[Aβ]/Ki[α-Syn]). Flutafuranol, flutemetamol, and PiB have 6-OH benzothiazoles motifs, and these had similar affinity and selectivity for Aβ fibrils and α-Syn fibrils. Florbetapir, a compound with a stilbene motif, also bound both types of fibrils.

A ^11^C isotopologue of BMB has been used to image myelin basic protein by PET,^12^ with further use as an intraoperative fluorescent agent for highlighting of nerves.^16^

The 5 Aβ imaging compounds have a narrow range of physical propertes (MW’s, LogP, tPSA, and cLogP) as indicated by their means, standard deviations, and coefficients of variation (SD/mean) tabulated at the bottom of Table 2.

## Discussion

For the series of BF-227-like compounds (Figure 1), increasing the length of R, either as the length of hydrocarbon chain (#2 through #6) or as the number of oxyethylene groups (#7 through #10), produced minimal increases in K_i_ and minimal effects on selectivity (K_i_ α-Syn/ K_i_ Aβ). The minimal effect of R size on K**_i_** is consistent with a model where the 2 rings of BF-227 (and our BF-227-like compounds) bind in a pocket of a β-sheet-based fibril, with the side chain relatively uninvolved.^17-19^ However, molecular docking studies have concluded that BF-227 can bind to a core binding site with an Aβ fibril.^20^

Our data indicate the difficulty in obtaining a highly selective α-Syn imaging agent binding to the common β-sheet structure of α-Syn and Aβ fibrils. First, all 5 of the reference PET imaging agents bound both Aβ and α-Syn fibrils, with higher affinity for Aβ fibrils. Second, BMB, which was developed as an imaging agent for mylein basic protein, had a K_i_ of 76 nM for Aβ fibrils which was not significantly different from the Aβ imaging agent PiB (Ki of 77 nM). In addition, a large literature with fluorescent and birefringent probes like thioflavin S, thioflavin T, and Congo red indicates they bind to the β-sheet motifs contained within many different proteins.

Removal of the 6-OH group on the benzoxazole ring and replacement with an iodine or fluorine (Table 1) reduced the affinity of BF-227 derivatives for both α-Syn and Aβ fibrils. None of the BF-227-like compounds had suffient α-Syn selectivity to be used for an α-Syn imaging agent. However, fluorine-bearing compounds # 13, 14, and 15 showed a modest improvement in α-Syn selectivity, most notably #15 which had considerable (5.1-fold) preference for α-Syn fibrils. A fluorobenzoxazole fragment might be employed in the design of future focused libraries employing fluorobenzoxazole fragments produced with the goals of obtaining a more α-Syn selective compound with affinities and molecular properties similar to existing clinical imaging agents.^21-22^ An advantage of future imaging agents using the fluorobenzoxazole fragment is the possiblilty of an ^18^F isotopologue for PET imaging. In addition, compounds containing fluorobenzoxazoles (or the closely related fluorobenzothiazoles) might offer an approach to find in vitro tools to enable better characterization of binding site densities in tissues of interest. Worth noting, benzoxazoles and benzothiazoles are often used in the design of compounds binding amyloid targets,^23-25^ thus supporting this strategy for the identification of potential novel α-Syn imaging agents.

## Supplemental Material

Supplementary_Data - The Binding of BF-227-Like Benzoxazoles to Human α-Synuclein and Amyloid β Peptide Fibrils Click here for additional data file. Supplementary_Data for The Binding of BF-227-Like Benzoxazoles to Human α-Synuclein and Amyloid β Peptide Fibrils by Lee Josephson, Nancy Stratman, YuTing Liu, Fang Qian, Steven H. Liang, Neil Vasdev, and Shil Patel in Molecular Imaging

## References

[1] Eberling JL Dave KD Frasier MA Alpha-synuclein imaging: a critical need for Parkinson’s disease research. J Parkinsons Dis. 2013;3(4):565–567. 24192754 10.3233/JPD-130247

[2] Vernon AC Ballard C Modo M Neuroimaging for Lewy body disease: is the in vivo molecular imaging of alpha-synuclein neuropathology required and feasible? Brain Res Rev. 2010;65(1):28–55. 20685363 10.1016/j.brainresrev.2010.05.006

[3] Mathis CA Lopresti BJ Ikonomovic MD Klunk WE Mathis CA Small-molecule PET tracers for imaging proteinopathies. Semin Nucl Med. 2017;47(5):553–575. 28826526 10.1053/j.semnuclmed.2017.06.003 PMC5657567

[4] Kudo Y Okamura N Furumoto S , et al. 2-(2-[2-dimethylaminothiazol-5-yl]ethenyl)-6-(2-[fluoro]ethoxy)benzoxazole: a novel PET agent for in vivo detection of dense amyloid plaques in Alzheimer’s disease patients. J Nucl Med. 2007;48(4):553–561. 17401091 10.2967/jnumed.106.037556

[5] Fodero-Tavoletti MT Mulligan RS Okamura N In vitro characterisation of BF227 binding to alpha-synuclein/Lewy bodies. Eur J Pharmacol. 2009;617(1-3):54–58. 19576880 10.1016/j.ejphar.2009.06.042

[6] Volpicelli-Daley LA Luk KC Lee VM Addition of exogenous alpha-synuclein preformed fibrils to primary neuronal cultures to seed recruitment of endogenous alpha-synuclein to Lewy body and Lewy neurite-like aggregates. Nat Protoc. 2014;9(9):2135–2146. 25122523 10.1038/nprot.2014.143 PMC4372899

[7] Cheng Y Prusoff WH Relationship between the inhibition constant (K1) and the concentration of inhibitor which causes 50 per cent inhibition (I50) of an enzymatic reaction. Biochem Pharmacol. 1973;22(23):3099–3108. 4202581 10.1016/0006-2952(73)90196-2

[8] Vlassenko AG Benzinger TL Morris JC PET amyloid-beta imaging in preclinical Alzheimer’s disease. Biochim Biophys Acta. 2012;1822(3):370–379. 22108203 10.1016/j.bbadis.2011.11.005 PMC3264790

[9] Kayed R Glabe CG Conformation-dependent anti-amyloid oligomer antibodies. Methods Enzymol. 2006;413:326–344. 17046404 10.1016/S0076-6879(06)13017-7

[10] Roberti MJ Folling J Celej MS Bossi M Jovin TM Jares-Erijman EA Imaging nanometer-sized alpha-synuclein aggregates by superresolution fluorescence localization microscopy. Biophys J. 2012;102(7):1598–1607. 22500760 10.1016/j.bpj.2012.03.010 PMC3318128

[11] Santa-Maria I Perez M Hernandez F Avila J Moreno FJ Characteristics of the binding of thioflavin S to tau paired helical filaments. J Alzheimers Dis. 2006;9(3):279–285. 16914838 10.3233/jad-2006-9307

[12] Stankoff B Wang Y Bottlaender M Imaging of CNS myelin by positron-emission tomography. Proc Natl Acad Sci U S A. 2006;103(24):9304–9309. 16754874 10.1073/pnas.0600769103 PMC1482605

[13] Ng KP Pascoal TA Mathotaarachchi S Monoamine oxidase B inhibitor, selegiline, reduces 18F-THK5351 uptake in the human brain. Alzheimers Res Ther. 2017;9(1):25. 28359327 10.1186/s13195-017-0253-y PMC5374697

[14] Harada R Ishiki A Kai H Correlations of 18F-THK5351 PET with post-mortem burden of tau and astrogliosis in Alzheimer’s disease. J Nucl Med. 2018;59(4):671–674. 28864633 10.2967/jnumed.117.197426

[15] Saint-Aubert L Lemoine L Chiotis K Leuzy A Rodriguez-Vieitez E Nordberg A Tau PET imaging: present and future directions. Mol Neurodegener. 2017;12(1):19. 28219440 10.1186/s13024-017-0162-3 PMC5319037

[16] Gibbs-Strauss SL Nasr KA Fish KM Nerve-highlighting fluorescent contrast agents for image-guided surgery. Mol Imaging. 2011;10(2):91–101. 21439254 PMC4386639

[17] Biancalana M Makabe K Koide A Koide S Molecular mechanism of thioflavin-T binding to the surface of beta-rich peptide self-assemblies. J Mol Biol. 2009;385(4):1052–1063. 19038267 10.1016/j.jmb.2008.11.006 PMC2664162

[18] Groenning M Binding mode of Thioflavin T and other molecular probes in the context of amyloid fibrils-current status. J Chem Biol. 2010;3(1):1–18. 19693614 10.1007/s12154-009-0027-5 PMC2816742

[19] Krebs MR Bromley EH Donald AM The binding of thioflavin-T to amyloid fibrils: localisation and implications protein particulates: another generic form of protein aggregation? J Struct Biol. 2005;149(1):30–37. 15629655 10.1016/j.jsb.2004.08.002

[20] Murugan NA Halldin C Nordberg A Laangstroem B Aagren H The culprit is in the cave: the core sites explain the binding profiles of amyloid-specific tracers. J Phys Chem Lett. 2016;7(17):3313–3321. 27498616 10.1021/acs.jpclett.6b01586

[21] Schuffenhauer A Ruedisser S Marzinzik AL Library design for fragment based screening. Curr Top Med Chem. 2005;5(8):751–762. 16101415 10.2174/1568026054637700

[22] Kumar A Voet A Zhang KY Fragment based drug design: from experimental to computational approaches. Curr Med Chem. 2012;19(30):5128–5147. 22934764 10.2174/092986712803530467

[23] Noel S Cadet S Gras E Hureau C The benzazole scaffold: a SWAT to combat Alzheimer’s disease. Chem Soc Rev. 2013;42(19):7747–7762. 23793644 10.1039/c3cs60086f

[24] Razavi H Powers ET Purkey HE Design, synthesis, and evaluation of oxazole transthyretin amyloidogenesis inhibitors. Bioorg Med Chem Lett. 2005;15(4):1075–1078. 15686915 10.1016/j.bmcl.2004.12.022

[25] Eckroat TJ Mayhoub AS Garneau-Tsodikova S Amyloid-beta probes: review of structure-activity and brain-kinetics relationships. Beilstein J Org Chem. 2013;9:1012–1044. 23766818 10.3762/bjoc.9.116 PMC3678428

