# Supplementary material for: The Binding of BF-227-Like Benzoxazoles to Human α-Synuclein and Amyloid β Peptide Fibrils
Source: Mol Imaging. 2018 Sep 14;17:1536012118796297. doi: 10.1177/1536012118796297 (PMC6144582; doi:10.1177/1536012118796297)
Supplement: Supplementary_Data - The Binding of BF-227-Like Benzoxazoles to Human α-Synuclein and Amyloid β Peptide Fibrils [file Supplementary_Data.pdf]

Supplementary Data

Title: The Binding of BF-227 and BF-227-like Benzoxazoles to Alpha-synuclein and Amyloid Beta Peptide Fibrils

Analysis of [^{3}H]BF-227 binding to human α-Syn and Aβ_{1-42} fibrils. Values are expressed as means ± SD.

Table S1: K_D and B_{max} for [^{3}H]BF-227 binding to α-Syn and Aβ_{1-42} fibrils				
Fibril	K_D (nM)	Bmax (pmol/nmol)	Mean K_D (nM)	Mean B_{max} (pmol/nmol)
α-Syn	47.7	13.5	46.0 ± 2.8	12.7 ± 1.1
	43.8	12.0		
Aβ	18.5	20.7	15.7 ± 3.1	19.4 ± 2.7
	13.2	16.3		
	15.4	21.3		
